# The Effects of Nurses’ Perception of the Older Adults and Work Stress on Nursing Competency of Nurses Who Care for Older Adult Patients at General Hospital

**DOI:** 10.3390/ijerph20032095

**Published:** 2023-01-23

**Authors:** Hwajin Lee, Minkyung Gu, Sohyune Sok

**Affiliations:** 1 Department of Nursing, Graduate School, Kyung Hee University, Seoul 02447, Republic of Korea; 2 Department of Nursing, College of Science and Technology, Daejin University, Pocheon-si 11159, Republic of Korea; 3 College of Nursing Science, Kyung Hee University, Seoul 02447, Republic of Korea

**Keywords:** aged, perception, work stress, nursing competency, nurse

## Abstract

The older adult population is rapidly increasing in South Korea, and hospitalization at general hospitals is increasing too. Therefore, nurses working at general hospitals need the nursing competency for older adult patients. The study was conducted to examine the effects of nurses’ perception of the older adults and work stress on the nursing competency of nurses at a general hospital, South Korea. A cross-sectional, descriptive correlational design was employed. Participants were a total of 136 nurses working at a general hospital located in Seoul, South Korea. Measures used in the study were the study participants’ general characteristics survey, Korean version of the Attitude Toward Old People Scale (KAOPS), the work stress scale, and the nursing competency scale. Data were collected from February to March, 2021. The regression model was statistically significant, and the explanatory power of the regression model was 33%. The significant factors affecting nursing competency were education level, perception of the older adults, and work stress. The greatest affecting factor was education level, followed by perception of the older adults and work stress in order. Nurses caring for older adult patients at general hospitals should pay attention to affecting factors to help improve the nursing competency in clinical practice. Managers should improve relevant policies to ensure that nurses have more opportunities to participate in the practical training of older adult care and explore effective training methods to improve the nurses’ perception of older adults.

## 1. Introduction

The rapid increase of the older adult population in South Korea, which accounted for 15.7% of the total population in 2020, is expected to account for 20.3% by 2025, thus leading to a super-aged society. In addition, in South Korea, the older adult population over 65 years of age continues to increase, accounting for 22.8% of the total households, with 49.6% expected to become older adult households by 2047 [1]. The increase in the older adult population like this resulted in the increase in the utilization rate of health care services by the older adults with chronic diseases [2,3]. In South Korea, among the older adult population over 65 years of age, the average number of visits to a medical institution in 1 month was 2.4, and it was found that more than 77.4% of the older adult population received treatment at a medical institution at least once [4]. As the number of older adult people visiting medical institutions increases in South Korea, so does the number of encounters with older adult patients and their caregivers, and the work of nurses who provide nursing services [5,6]. In this regard, the increase in the work of nurses may lead to a negative perception of the older adults, which may be a factor that increases work stress on nursing [3,5].

Rapid population aging has many impacts on aging perception, age prejudice and discrimination, and consequent public health and policy, resulting in environmental and social issues [5,7,8]. In the time of industrialization and information, negative perceptions and prejudices about the aging and aged are converted by the extension of human lifespan due to medical and scientific development [2,3,5,8]. Social, cultural and environmental issues are influencing public health care policies along with national policies [3,5,8,9,10]. Along with the rapidly growing older adult population, the health care community will need to prepare for the rapidly growing older adult population as health care consumers [10]. In particular, in the current situation where the number of older adult patients is increasing in general hospitals, the perception and attitude of medical personnel and nurses toward the older adults will greatly affect the improvement of the quality of nursing care [5,9].

On the other hand, it is necessary to recognize that the older adults are not only biologically declining and reduced as they age, but that they can improve their life satisfaction and quality of life by maintaining and managing their biological health through the normal aging process [7,8,9]. The process of getting older, that is, aging, can be seen as a process of profound maturation in terms of social and cultural aspects, although there is a biological aspect of physical function decline [2,5,10]. This perception of aging is a correct understanding of human beings, and it can be seen as an important perception of aging, especially for medical personnel and nurses who deal with human life [5,6,10,11,12]. Therefore, as the number of older adult patients is increasing not only in geriatric wards but also in general wards, health care professionals including nurses need education and training in knowledge and perception of the elderly [8,9]. However, since 2008, when the long-term care law was enacted in South Korea, education and training for the older adults have been implemented, but they are insufficient [6,8,9]. Lack of perception of the older adults gives rise to a negative effect on the nurses’ therapeutic nursing performance for the older adults [10,11], thus leading to work stress, and ultimately may act as a factor that lowers the nursing competency of nurses who need to perform professional nursing [10,12]. From the universal perception of the older adults as biologically weak and declining beings [7,8] to beings with a healthy physical function maintained through health care and improving life satisfaction and quality of life, to beings as socially culturally mature human, transition of the perception of the aging process is needed [8,9,10]. Such perception by health care professionals, including nurses, increases respect for the older adults and recognizes the value of the older adults, which will improve the quality of care for the older adults.

Nursing competency requires comprehensive knowledge and skills to effectively perform nursing practice [2,13], including values, attitudes, and beliefs that reflect nursing skills [8]. It was also found to be an important factor in the quality management of nursing, and to help in performing better nursing care in duties [13,14]. Based on the results of the previous studies, in this trend of an increasing older adult population, the nurses’ nursing competency for the older adults was closely related to various human resources and work characteristics [10,14,15]. Above all, nursing competency can affect the nursing work productivity for the older adults [10,14].

Therefore, in view of the increasing number of older adults hospitalized in general hospitals, it is considered to be very meaningful to examine the nurses’ perception of the older adults and work stress, and to understand the effect on the nursing competency of nurses who care for older adult patients admitted to general hospitals. The purpose of this study was to examine the effect of perception of the older adults and work stress on the nursing competency of nurses. The aims of the study were: (1) to identify the general characteristics of the study participants; (2) to examine the degrees of perception of the older adults, work stress, and nursing competency; (3) to examine the differences on perception of the older adults, work stress, and nursing competency according to the general characteristics of the study participants; (4) to examine the correlations among perception of the older adults, work stress, and nursing competency; (5) to examine the effects of the perceptions of the older adults and work stress on nursing competency.

## 2. Material and Methods

### 2.1. Study Design and Participants

A cross-sectional, descriptive correlational design was employed. The participants of this study were a total of 136 nurses working at a general hospital located in Seoul. They were working at unit caring general adult patients including elderly patients, and recruited using convenience sampling. As the included criteria, nurses with more than 1 year of work experience caring for elderly patients were selected as those who understood the purpose of this study and agreed to voluntarily participate.

The number of participants in this study was calculated with a significance level of 0.05 and an effect size of 0.25 to secure 80% or more of statistical power for multiple regression analysis of the G Power 3.1.2 program [16]. Therefore, the appropriate sample size was 123 persons. Considering the dropout rate of about 15%, 140 people were asked to fill out a questionnaire. The number of samples in this study was 133 (95%), excluding 7 who did not respond or omitted questionnaires, and were finally used for data analysis.

### 2.2. Measures

Study participants’ general characteristics included gender, age, marital status, educational level, position, and clinical experience. This consisted of a total of 6 items.

For the perception scale for the older adults, the Attitude Toward Old People Scale (KAOPS) developed by Kogan [17], modified and supplemented by Lee [18], was used in this study. This scale examining the nurses’ perception of the older adults has a total of 34 items, and 17 items such as the older adults’ residential environment, cognitive type, competence, appearance, personality, independence, intergenerational interpersonal relationships, and discomfort with the older adults are each paired. Each item is scored on a 6-point Likert scale with 1 point for ‘strongly disagree’ and 6 points for ‘strongly agree’. The score ranges from a minimum of 34 to a maximum of 204, and the higher the score, the more positive the perception of the older adults. In Lee’s [18] study, the reliability of the scale was Cronbach’s α = 0.79, and in this study, the reliability of the scale was Cronbach’s α = 0.73.

The work stress scale is a scale that measures the stress experienced by nurses while performing their duties. In this study, the scale developed by Gu and Kim [19] was used. This scale has a total of 43 items, including nursing tasks 6 items, role conflicts 5 items, expertise 4 items, conflicts with doctors 3 items, interpersonal relationships 6 items, psychological burden 3 items, nurse treatment 5 items, work schedule 7 items, and related to patients and caregivers 4 items. As a 5-point Likert scale, the score ranges from a minimum of 43 to a maximum of 215, and the higher the score, the higher the nurse’s work stress. In the study of Gu and Kim [19], the reliability of the scale was Cronbach’s α = 0.94, and in this study, the reliability of the scale was Cronbach’s α = 0.96.

Nursing competency scales developed by Kim [20] were used. This scale has a total of 53 questions, including nursing practice skills 9 questions, safety management ability 5 questions, nursing unit management ability 10 questions, critical thinking ability 5 questions, interpersonal relationship ability 6 questions, leadership ability 5 questions, education and counseling ability 5 questions, 4 questions on ethical practice ability, and 4 questions on professional development ability. As a 5-point Likert scale, the score ranges from the lowest point of 53 to the highest point of 265, and the higher the score, the higher the nurse’s nursing competency. In the study of Kim [20], the reliability of the scale was Cronbach’s α = 0.98, and in this study, the reliability of the scale was Cronbach’s α = 0.97.

### 2.3. Procedures

This study was conducted from February to March, 2021 after receiving approval from the Institutional Research Board of S General Hospital located in Seoul. After the researchers visited S General Hospital and received approval from the Ministry of Nursing, a questionnaire was distributed to nurses with more than one year of working experience, explaining the purpose of the study and voluntarily agreeing to participate. One nurse took about 30 min to fill out the questionnaire, and after distributing the questionnaire, the researcher thoroughly reviewed the collected data for any abnormalities.

### 2.4. Statistical Analysis

The collected data were analyzed using SPSS Windows Version 25.0 Program. Reliability coefficients were calculated using Cronbach’s-α, which is an internal consistency test, for the subject’s reliability of the measurement scale. The general characteristics of the study participants were analyzed in terms of frequency and percentage using descriptive statistics. The degrees of perception of the older adults, work stress, and nursing competency were analyzed using descriptive statistics and frequency analysis as the mean and standard deviation. Independent t-test and ANOVA were used to analyze differences in the perception of the older adults, work stress, and nursing competency according to the general characteristics of the study subjects, and all significant variables were analyzed using the Scheffe post hoc test. Correlations among major variables were examined using Pearson’s correlation coefficient. Stepwise multiple regression analysis was used for all significant variables in order to examine the effects of the study subjects’ perceptions of the older adults and work stress on nursing competency.

### 2.5. Ethical Considerations

In ethical considerations, after this study was approved by the Institutional Research Board of Seoul hospital (IRB No. SEOUL 2021-01-006-001), voluntary written consent was obtained from the study participants for data collection, and it was revealed that the collected data would not be used for any purpose other than the research purpose. In addition, it was explained that the participants’ anonymity and confidentiality were communicated to them, explaining that they can withdraw at any time during the study if they do not wish to.

## 3. Results

### 3.1. General Characteristics of the Study Participants

Participants in this study were four males (3%) and 129 females (97%). The average age was 30.2 years, and 61 patients (45.9%) between the ages of 25 and 29 accounted for the majority. As for education level, 124 (93.2%) had a university degree. As for the total clinical experience, 62 patients (46.6%) with less than 5 years accounted for the majority, followed by 37 patients (27.9%) between 5 and 9 years (Table 1).

### 3.2. Levels of Perception of the Older Adults, Work Stress, and Nursing Competency

Looking at the degrees of perception of the older adults, work stress, and nursing competency, the perception of the older adults was 117.58 points on average total score. The average total score for work stress was 169.88, and nursing competency was average total score of 188.98.

### 3.3. Differences on Perception of the Older Adults, Work Stress, and Nursing Competency according to the General Characteristics of the Study Participants

In this study, the degree of perception of the older adults according to the general characteristics of the study participants showed significant difference in marital status (F = −2.988, *p* = 0.003). This can be seen that nurses married had the more positive perception of the older adults. The degree of work stress according to the general characteristics of the study participants showed significant differences in age (F = 4.839, *p* = 0.001), position (F = 2.434, *p* = 0.049), and clinical experience (F = 4.896, *p* = 0.001). This can be seen that the general nurses between the ages of 30 and 34, with clinical experience of 5 to 14 years, had the higher work stress. The degree of nursing competency according to the general characteristics of the study participants showed significant differences in age (F = 8.875, *p <* 0.001), marital status (F = −4.261, *p* < 0.001), education (F = 2.585, *p* = 0.011), position (F = −5.455, *p* = 0.001), and clinical experience (F = 10.568, *p <* 0.001). Nursing competency was relatively high for senior nurses who were 40 years of age or older, married, and graduated from university. Nurses with more than 20 years of clinical experience had higher nursing competency (Table 2).

### 3.4. Correlations among Perception of the Older Adults, Work Stress, and Nursing Competency

The correlations among the perception of the older adults, work stress and nursing competency did not show significant correlations. However, there was a positive correlation between the perception of the older adults and nursing competency (*γ* = 0.303, *p* < 0.001).

### 3.5. Factors Affecting Nursing Competency

In order to determine whether this study is suitable for regression analysis, the assumptions of the regression model were verified. First, the autocorrelation of errors was tested with Durbin–Watson to verify the independence of the residuals. As a result, the statistic was 1.772, which satisfied the assumptions of the regression equation. In addition, the tolerance of multicollinearity was 0.184 to 0.802, which was over 0.10, and the variance inflation factor (VIF) was 1.00 to 5.43, which was not greater than 10, so all variables did not have a problem with multicollinearity.

The effects on nurses’ nursing competency were analyzed by using the stepwise multiple regression. As a result, it was found to be statistically significant in the multiple regression model (F = 9.165, *p* < 0.001), and the statistically significant variables were education level (β = −0.17, *p* = 0.034), perception of the older adults (β = 0.16, *p* = 0.032), and work stress (β = 0.13, *p* = 0.016), and the explanatory power of the regression model was 33%. Through this study, the most important variable influencing the nurses’ nursing competency was education level, followed by perception of the older adults and work stress, in that order (Table 3).

## 4. Discussion

As a result of analyzing the nurses’ perception of the older adults in this study, the perception of the older adults showed a moderate score, which was consistent with the results of the study of Lee [18] showing a neutral attitude toward the older adults. The reason why the study subjects were not positive about the older adults was consistent with the findings of Kim and Lee [8], reporting that when medical personnel encounter the older adults, they have health problems or they are at their most debilitating. For this reason, the medical personnel have fewer positive experiences with the older adults, and since the medical personnel are more concerned with treatment rather than the nursing needs of the older adult patients, it is possible for them to have relatively negative attitudes [5,7,11]. The nurses’ negative attitude toward the older adults can negatively affect the quality of nursing received by the patients [8,11,18]. In order to provide quality nursing to older adults and increase nursing competency, the nurses should have a positive view of the older adults as a person with the heart of caring for their family, rather than the view the older adults as foolish, sick, and old individuals [2,6,11].

As a result of analyzing the level of work stress of nurses, it was found that work stress was higher than average, which was consistent with the study results of Shin [21]. According to the results of these previous studies, the stress from patients and caregivers was the highest among the stress experienced by nurses while working, followed by stress from conflict with the doctors, stress from psychological burden, and stress from nursing work. Although the perception and treatment of patients and caregivers towards nurses have significantly improved, it is still lacking. As such, nurses are under a lot of mental and physical stress, and in order to control this, it is necessary to prepare alternatives to improve the perception of patients and caregivers.

As a result of analyzing the level of nursing competency of nurses, it was found that nursing competency was evaluated to be higher than the intermediate level, which was consistent with the result of the previous study by Yoo [22]. It shows that the nurses generally evaluate their competency at a higher level. This is presumed to be due to the increasing number of highly educated nurses whose work has become more specialized and more knowledgeable due to the development of science and medicine, and the nursing competency is highly evaluated [3,10,15,22]. In this regard, further research will be able to clarify the justifications grounds for the results of this study.

In this study, the nurses’ perception of the older adults was more positive when they were married, which was similar to the study of Lee et al. [3]. Recently, due to the nuclear family, the contact between the older adults and the younger generation is decreasing, the age group is relatively young, and the opportunity for unmarried nurses to face the older adults is decreasing [2,23]. On the other hand, it is thought that this is because married nurses have a view of accepting the older adults as a person rather than a foolish, sick, and old individual when recognizing the older adults due to the strong family role that is formed based on a high level of trust with the family [9,23].

Nurses’ work stress was higher in general nurses aged 30–34 and with 5–14 years of clinical experience. This was similar to the research results of Nam [24], Nam and Cho [25], who reported that general nurses aged 35 years or older and with more than 15 years of clinical experience experienced low work stress regardless of the patient’s situation and environment. However, in this study, it was found that young nurses under the age of 29 and nurses with less than 5 years of clinical experience had lower work stress, which was different from previous research results [24,25]. It is speculated that this is a changed phenomenon due to the personality and temperament of young people of the MZ generation these days. These results need to be more clearly verified through continuous follow-up studies. Due to the rapidly changing medical environment and the ever-changing situation of patients and caregivers, nurses may react more sensitively and have higher work stress [9,25]. In addition, while nursing for the older adults, nurses may have a lot of emotional labor to understand themselves and the older adults and control their minds, so the burden of caring for the older adult patients may be high and work stress may be high [3,10,15]. Therefore, it is necessary to introduce strategies and intervention programs that can reduce the emotional labor management and work stress of nurses who care for older adult patients [7,26].

The nursing competency of nurses was relatively higher among the charge nurses who were 40 years of age or older, married, and graduated from a university. This was similar to the study result of Kim et al. [27] in that nurses with more than 20 years of clinical experience have a higher nursing competency. These results support the study of Kim and Gu [13] in that the better the treatment of nurses (clinical experience, position, and average monthly salary), the higher the nurse’s competency, depending on the size of the hospital organization. It is thought that systematic measures within the hospital organization should be prepared in detail to improve the nursing competency that can lead to the resilience of nurses for each department.

The nurses’ perception of the older adults and nursing competency were found to have a positive correlation, which was similar to the study results of Darcy [26] and Kim and Kwon [5]. The more positive the perception of the older adults, the higher the level of nursing competency. Therefore, it was found that a positive perception of the older adults is necessary in order to improve the nursing competency for the older adults.

Finally, the result of analyzing the effect on the nurses’ nursing competency showed that the nurse’s education level, perception of the older adults, and work stress were found in that order. This can be said to support the fact that the nurses need to have a good understanding and empathy for the older adults, going beyond acquiring disease and biological expertise to meet the multidimensional health needs of the older adults [11,26]. In other words, the higher educational level of the nurse affects the higher nursing competency of the nurse. It was found that the nurses’ positive perception of the older adults and reduced work stress can improve the nursing competency for the older adults [2,8].

Based on the above results, nurses caring for older adult patients at general hospitals need to improve their positive perception of the elderly and reduce work stress to increase their nursing competency. Therefore, it is necessary to encourage the nurses to steadily develop a correct understanding of the aging process and the older adults through a specialized education on the older adults [5,8,11,14,28] so that improvement of perception of the older adults can be prioritized, with systematic and specific measures to reduce work stress during nursing intervention. It is necessary to provide a wide range of continuing education for the nurses in order to encourage a positive perspective on the older adults and operate a program within the hospital to cope with work stress that may occur while nursing for the older adults. In addition, hospitals need to improve perception for the older adults, provide sufficient rest, and replenish insufficient manpower in order to reduce work stress, and accurately diagnose and supplement problems in nursing care according to the establishment of a systematic and efficient nursing system for the older adults [14,29,30]. Managing work stress in nursing for the older adults is thought to contribute to improving the quality of nursing services provided to older adult patients. In addition, hospital managers should prepare specific strategies and measures to reduce the nurses’ work stress and strengthen nursing competency, and at the same time, improve their perception of the older adults and have follow-up management to check whether work stress is adequately controlled. For further research, a qualitative study is deemed necessary to understand and analyze the nurses’ perception of the older adults and work stress in their inner world more deeply. It is also necessary to develop an intervention program related to the perception of the older adults and work stress for nurses, and to conduct an experimental study to verify it.

This study has some limitations. The research on the nurses’ perception of the older adults and work stress is insufficient, and the subjects of this study were recruited using convenience sampling from one general hospital. As such, there are limitations to explain the effects of all nurses’ perception of the older adults and work stress on nursing competency due to the limitation in sampling. Therefore, it is necessary to conduct repeated and expanded studies in consideration of the sampling of future study subjects.

## 5. Conclusions

In conclusion, the more positive the perception of the older adults, the higher the level of nursing competency. Influencing factors on the nursing competency of nurses appeared in the order of education level, perception of the older adults, and work stress. The positive perception of older adult patients, who are rapidly increasing in general wards, will improve nursing competency and ultimately raise the quality of nursing care. A positive perception of the older adults can be achieved through education and training on the knowledge and understanding of the older adults. In addition, it is thought that it is important to seek and apply strategies and intervention methods that can reduce work stress in order to improve nursing competency of nurses.

This study is significant in that it mentions the need for specialized education on the older adults in order to improve the nurses’ positive perception of the older adults. It also provides fundamental data for developing an intervention program that can reduce the nurses’ work stress and improve their nursing competency.

## Figures and Tables

**Table 1:** General characteristics of the study participants.

Characteristics	*n*	%
Gender		
Male	4	3.0
Female	129	97.0
Age		
<25	19	14.3
25–29	61	45.9
30–34	31	23.3
35–39	10	7.5
≥40	12	9.0
Marital status		
Single	102	76.7
Married	31	23.3
Educational level		
≤University	124	93.2
≥Graduate school	9	6.8
Position		
General nurse	126	94.7
Charge nurse	7	5.3
Clinical experience (year)		
5>	62	46.6
5–9	37	27.9
10–14	20	15.0
15–19	6	4.5
20≤	8	6.0

**Table 2:** Differences on perception of the older adults, work stress, and nursing competency according to the general characteristics of the study participants.

Characteristics	Perception of the Older Adults		Work Stress		Nursing Competency	
	Mean ± SD	t or F (*p*) Scheffe	Mean ± SD	t or F (*p*) Scheffe	Mean ± SD	t or F (*p*) Scheffe
Gender		0.998 (0.390)		1.181 (0.321)		1.375 (0.259)
Male	110.00 ± 15.56		145.75 ± 41.91		162.00 ± 40.04	
Female	117.81 ± 10.41		170.63 ± 23.85		189.81 ± 32.63	
Age		1.593 (0.180)		4.839 (0.001 *) c > e		8.875 (<0.001 *) a < e
<25 ^a^	115.26 ± 8.44		159.74 ± 25.83		172.47 ± 41.26	
25–29 ^b^	116.26 ± 12.33		169.87 ± 23.31		179.57 ± 28.91	
30–34 ^c^	118.29 ± 8.76		182.45 ± 11.78		198.64 ± 24.16	
35–39 ^d^	121.70 ± 6.96		171.30 ± 27.68		204.90 ± 26.88	
≥40 ^e^	122.67 ± 9.53		152.33 ± 35.97		224.67 ± 26.96	
Marital status		−2.988 (0.003 *)		−0.586 (0.559)		−4.261 (<0.001 *)
Single	116.11 ± 10.35		169.19 ± 23.94		182.65 ± 31.31	
Married	122.42 ± 10.14		172.16 ± 27.30		209.81 ± 30.29	
Education		1.747 (0.083)		0.969 (0.334)		2.585 (0.011 *)
College	120.48 ± 11.68		175.48 ± 17.79		183.58 ± 31.31	
University	116.46 ± 10.25		170.71 ± 23.76		200.93 ± 28.94	
Position		−1.210 (0.269)		2.434 (0.049 *)		−5.455 (0.001 *)
General nurse	117.27 ± 10.46		171.79 ± 22.36		186.20 ± 31.24	
Charge nurse	123.14 ± 12.60		135.43 ± 39.18		239.00 ± 24.53	
Clinical experience (year)		2.301 (0.062)		4.896 (0.001 *) b,c > e		10.568 (<0.001 *) a < e
5> ^a^	116.03 ± 9.25		166.37 ± 26.03		175.45 ± 32.69	
5–9 ^b^	116.92 ± 13.70		176.30 ± 14.01		188.70 ± 26.36	
10–14 ^c^	121.05 ± 7.52		180.50 ± 22.30		206.50 ± 25.08	
15–19 ^d^	115.00 ± 7.69		168.00 ± 13.14		217.33 ± 14.53	
20≤ ^e^	125.88 ± 8.43		142.25 ± 39.75		230.00 ± 27.02	

* *p* < 0.05. a, b, c, d, e shows the post hoc test using Scheffe.

**Table 3:** Factors affecting nursing competency.

Variables	B	S.E	β	t	*p*	DW	Tolerance	VIF
Gender	2.01	1.98	0.04	1.21	0.218	1.772	0.433	1.87
Age (year) ˡ	−1.27	1.02	−0.26	−1.25	0.215		0.184	4.22
Marital status	−0.79	7.16	−0.01	−0.11	0.912		0.446	1.27
Education	−11.00	5.19	−0.17	−2.14	0.034 *		0.748	1.24
Position	19.83	16.12	0.14	1.23	0.221		0.322	2.26
Clinical experience ˡ (years)	0.21	0.11	0.51	1.86	0.065		0.186	5.43
Perception of the older adults	0.50	0.23	0.16	2.16	0.032 *		0.802	1.00
Work stress	0.17	0.10	0.13	1.63	0.016 *		0.624	1.18
R^2^ = 0.35, Adjusted R^2^ = 0.33, F = 9.165, *p* < 0.001 *								

ˡ Dummy variable: Age (Reference ≥ 40), Clinical experience (Reference 20≤); * *p* < 0.05.

## Data Availability

No new data were created or analyzed in this study. Data sharing is not applicable to this article.
